# Application of the Fluorescence-Activating and Absorption-Shifting Tag (FAST) for Flow Cytometry in Methanogenic Archaea

**DOI:** 10.1128/aem.01786-22

**Published:** 2023-03-15

**Authors:** Norman Adlung, Silvan Scheller

**Affiliations:** a School of Chemical Engineering, Department of Bioproducts and Biosystems, Aalto University, Espoo, Finland; University of Nebraska-Lincoln

**Keywords:** Methanogenic archaea, fluorescence, flow cytometry, FAST, *Methanosarcina*

## Abstract

Methane-producing archaea play a crucial role in the global carbon cycle and are used for biotechnological fuel production. Methanogenic model organisms such as Methanococcus maripaludis and Methanosarcina acetivorans have been biochemically characterized and can be genetically engineered by using a variety of existing molecular tools. The anaerobic lifestyle and autofluorescence of methanogens, however, restrict the use of common fluorescent reporter proteins (e.g., GFP and derivatives), which require oxygen for chromophore maturation. Recently, the use of a novel oxygen-independent fluorescent activation and absorption-shifting tag (FAST) was demonstrated with *M. maripaludis*. Similarly, we now describe the use of the tandem activation and absorption-shifting tag protein 2 (tdFAST2), which fluoresces when the cell-permeable fluorescent ligand (fluorogen) 4-hydroxy-3,5-dimethoxybenzylidene rhodanine (HBR-3,5DOM) is present. Expression of tdFAST2 in *M. acetivorans* and *M. maripaludis* is noncytotoxic and tdFAST2:HBR-3,5DOM fluorescence is clearly distinguishable from the autofluorescence. In flow cytometry experiments, mixed methanogen cultures can be distinguished, thereby allowing for the possibility of high-throughput investigations of the characteristic dynamics within single and mixed cultures.

**IMPORTANCE** Methane-producing archaea play an essential role in the global carbon cycle and demonstrate great potential for various biotechnological applications, e.g., biofuel production, carbon dioxide capture, and electrochemical systems. Oxygen sensitivity and high autofluorescence hinder the use of common fluorescent proteins for studying methanogens. By using tdFAST2:HBR-3,5DOM fluorescence, which functions under anaerobic conditions and is distinguishable from the autofluorescence, real-time reporter studies and high-throughput investigation of the mixed culture dynamics of methanogens via flow cytometry were made possible. This will further help accelerate the sustainable exploitation of methanogens.

## INTRODUCTION

Methanogenic archaea are responsible for up to 70% of the methane emitted globally (1). Aside from their relevance to the global carbon cycle, methanogens show great potential for various biotechnological applications, such as production of biogas and biofuel (2), treatment of solids and sewage water (3), carbon dioxide utilization (4, 5), and storage of excess electricity via bioelectrochemical systems (6). Genetically tractable methanogens are exemplified by Methanococcus maripaludis and several *Methanosarcina* species. Genetic tools for these methanogens include plasmid-expression systems, genome modification via homologous recombination and CRISPR/Cas, inducible gene expression, and reporter systems (7–9).

The oxygen sensitivity of methanogens limits their use with common fluorescent proteins. Here, fluorescent proteins with a barrel-like structure (e.g., GFP and mCherry) require oxygen for chromophore maturation, and thus will not fluoresce under anaerobic conditions (10). The use of fluorescent proteins in methanogens is also hindered by the cellular autofluorescence (emission maximum at 480 nm), which originates from oxidized coenzyme F420 after excitation at 420 nm (11). Consequently, valuable molecular tools, such as protein localization via fluorescence microscopy, high-throughput measurement of protein accumulation via flow cytometry, and fluorescence-activated cell sorting (FACS), remain underexplored in methanogens. In recent years, novel tools that would replace oxygen-dependent fluorescent proteins under anoxic conditions have been developed by employing different mechanisms, e.g., flavin mononucleotide-based fluorescent proteins, fluorescence activating proteins that require reversible binding of a fluorogenic ligand, and self-labeling proteins that require covalent binding to nonfluorogenic ligands (12, 13). Recently, the use of a fluorescence activating protein was successfully demonstrated with *M. maripaludis* (14).

Fluorescence-activating proteins such as FAST (fluorescence-activating and absorption-shifting tag) and its improved version FAST2 are small, engineered proteins that fluoresce after binding to a fluorogenic ligand (fluorogen) (15, 16). FAST and FAST2 can be fused to a protein of interest, be expressed as a single protein or in tandem to yield a higher level of fluorescence (tdFAST/tdFAST2). Different fluorogens were developed; they are 4-hydroxybenzylidene rhodanine derivatives and determine the excitation/emission wavelength of the FAST:fluorogen fluorescene upon interaction (15, 16). FAST can also be used for *in vivo* studies of protein-protein interactions by fusing the N- and C-terminal regions to the proteins of interest (splitFAST) (17). Here, FAST:fluorogen fluorescence is detectable when the two proteins of interest interact, thereby bringing the N- and C-terminal regions of FAST in close proximity. In our present study, we apply the FAST reporter to the methanogenic model organism *M. acetivorans* and demonstrate its optimized use for making reliable flow cytometry measurements.

## RESULTS

### tdFAST2:HBR-3,5DOM fluorescence in methanogens.

**FIG 1:**
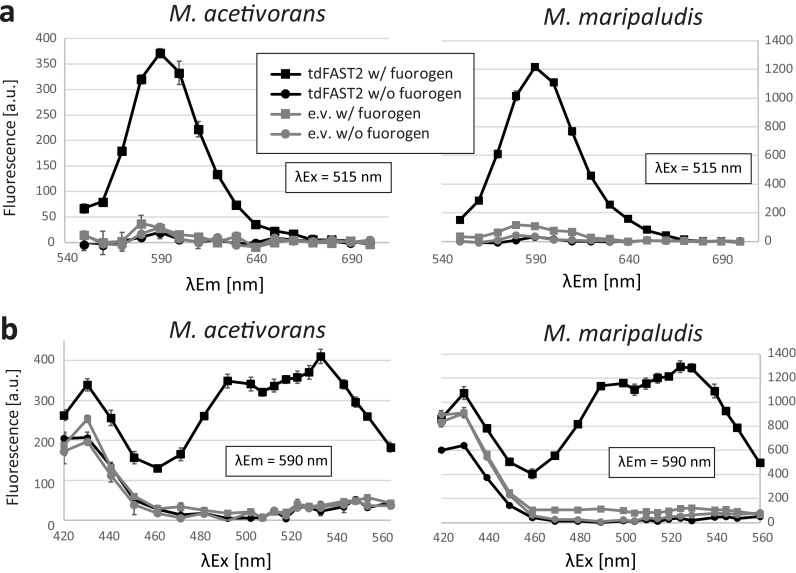
tdFAST2-expressing methanogens show a specific fluorescence in the presence of HBR-3,5DOM. Fluorescence of *M. acetivorans* and *M. maripaludis* cells is shown. (a) Fluorescence spectrum upon excitation at 515 nm. (b) Fluorescence at 590 nm when different excitation wavelengths are applied. Cells expressing tdFAST2 (tdFAST2) or control cells carrying an empty vector construct (e.v.) were analyzed in presence (w/ fluorogen) or absence (w/o fluorogen) of HBR-3,5DOM. Cells were in the stationary growth phase. Mean values and standard deviation of triplicates are shown.

Plasmid constructs for constitutive expression of tdFAST2 in *M. acetivorans* (pNB730::tdFAST2) and *M. maripaludis* (pMEV4::tdFAST2) were generated and transformed into each methanogen(for details, see Materials and Methods). Cells were also transformed with the original (empty) vectors and served as a control. For taking measurements, liquid cultures were analyzed using a microplate reader under aerobic conditions and a washing step was included to eliminate background fluorescence from the culture medium. In the presence of the HBR-3,5DOM fluorogen, tdFAST2-producing *M. acetivorans* and *M. maripaludis* cells showed a fluorescence peak around 590 nm after 515 nm excitation (Fig. 1a). In the absence of the fluorogen or tdFAST2, this fluorescence peak was not detected. Thus, tdFAST2:HBR-3,5DOM fluorescence is clearly distinguishable from the cellular autofluorescence. Further analysis showed that tdFAST2:HBR-3,5DOM fluorescence can be excited with a wide range of wavelengths, i.e., from 480 nm to 550 nm (Fig. 1b). This contrasts with the fluorogen HMBR (4-hydroxy-3-methylbenzylidene rhodamine), which has a rather narrow excitation range at around 490 nm (Fig. S1). In all subsequent experiments, excitation/emission wavelength were set at 515/590 nm for the detection of tdFAST2:HBR-3,5DOM fluorescence.

### Influence of cell density and fluorogen concentration.

**FIG 2:**
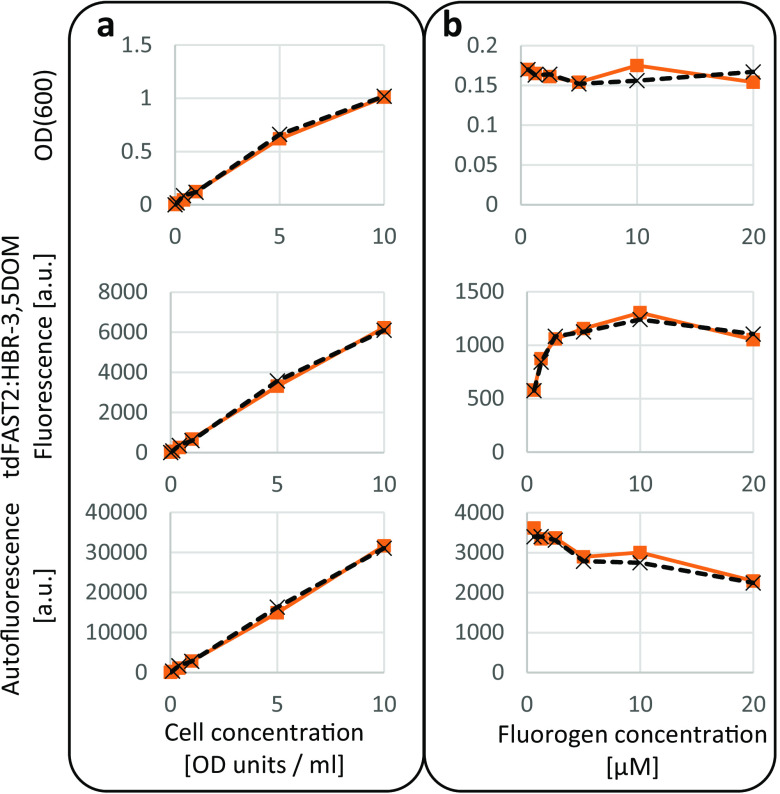
Wide ranges of cell concentration and fluorogen concentration can be used to measure tdFAST2:HBR-3,5DOM fluorescence in *M. acetivorans*. *M. acetivorans* cells expressing tdFAST2 were analyzed in the exponential growth phase. A microplate reader was used to measure OD(600), tdFAST2:HBR-3,5DOM fluorescence (λ_Ex_ = 515 nm/λ_Em_ = 590 nm), and autofluorescence (λ_Ex_ = 420 nm/λ_Em_ = 480 nm). (a) Correlation of fluorescence and cell-density when the fluorogen concentration (5 μM) is constant. (b) Influence of the fluorogen concentration on the fluorescence when the cell concentration is constant (1 OD unit/mL). Note that, due to different light path lengths, the OD(600) determined by the microplate reader (upper panel) is smaller than the actual cell concentration (OD units/mL) which was determined using a standard spectrophotometer. Values of duplicates are shown.

The influence of different cell and fluorogen concentrations on tdFAST2:HBR-3,5DOM fluorescence in *M. acetivorans* was studied. When the *M. acetivorans* cell concentration was increased, but the HBR-3,5DOM fluorogen concentration held constant at 5 μM (as recommended by the manufacturer), tdFAST2:HBR-3,5DOM fluorescence exhibited a linear increase (Fig. 2a). By comparison, measurement of autofluorescence and OD(600) measured by the microplate reader showed a similar increase (Fig. 2a), and thus could be used for normalizing the tdFAST2:HBR-3,5DOM fluorescence values within different cultures.

To determine an optimal fluorogen concentration, the HBR-3,5DOM fluorogen was varied from 0.625 to 20 μM, while the cell concentration was kept constant (1 OD unit/mL). Here, tdFAST2:HBR-3,5DOM fluorescence increased sharply to a concentration of 2.5 μM before plateauing out (Fig. 2b). Notably, higher fluorogen concentrations led to a decrease of autofluorescence but did not influence the OD(600) measured by the microplate reader. The autofluorescence decrease at higher fluorogen concentrations might be due to quenching from partially overlapping excitation spectra or due to a direct or indirect reduction of oxidized F420. Based on these results, 5 μM fluorogen was used for subsequent experiments. Overall, our findings show that the tdFAST2:HBR-3,5DOM interaction in *M. acetivorans* delivers a reliable source of fluorescence when either the cell concentration and fluorogen concentration is kept constant.

### tdFAST2:HBR-3,5DOM fluorescence during different growth phases.

**FIG 3:**
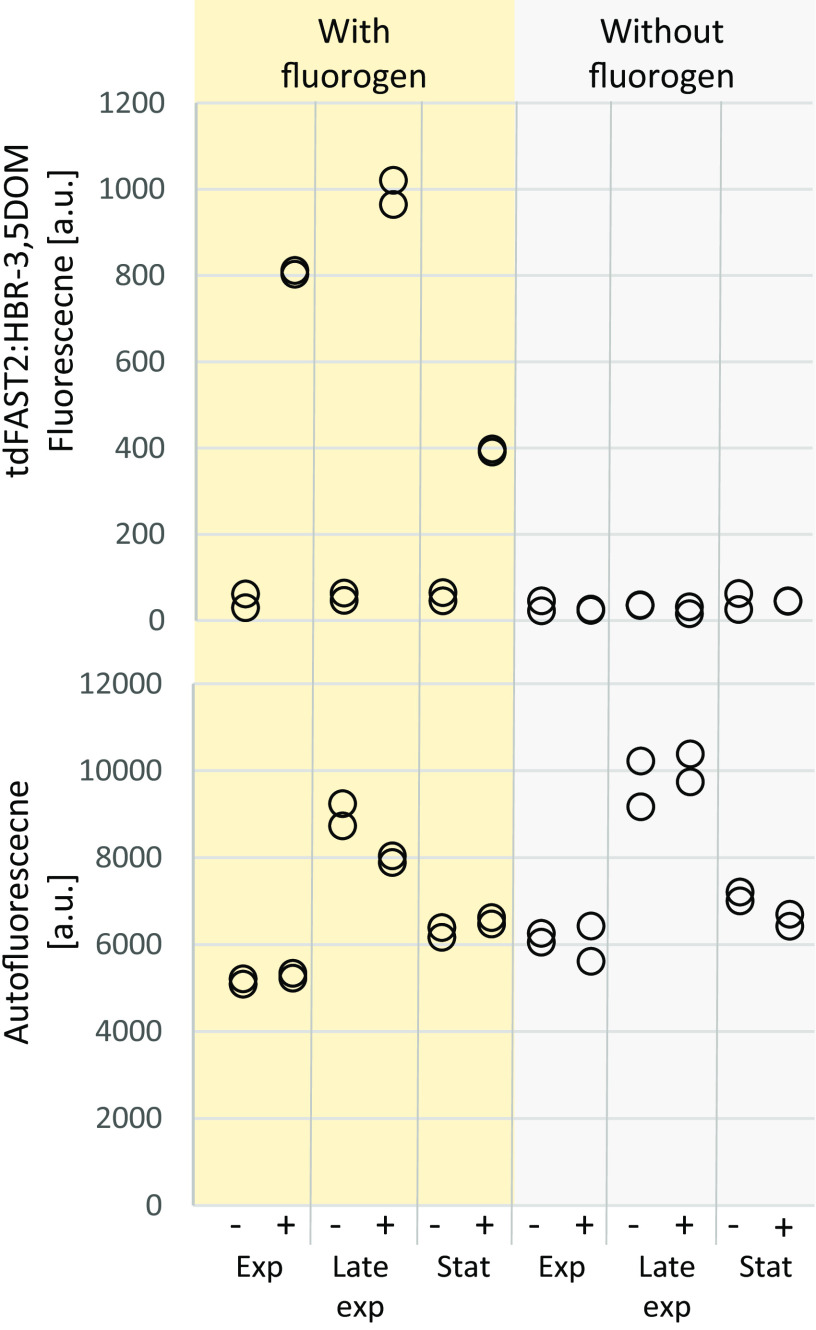
*M. acetivorans* fluorescence changes at different growth phases. *M. acetivorans* cells expressing tdFAST2 (+) or harboring an empty vector construct (–) were analyzed at exponential (Exp; OD[600] 0.3–0.6), late exponential (Late Exp; 0.8 to 1.1), and stationary growth (Stat; >1.1). tdFAST2:HBR-3,5DOM fluorescence (λ_Ex_ = 515 nm/λ_Em_ = 590 nm), and autofluorescence (λ_Ex_ = 420 nm/λ_Em_ = 480 nm) was measured. Technical duplicates are shown.

Monitoring protein accumulation over time is an important application of fluorescent reporter systems. Therefore, tdFAST2:HBR-3,5DOM fluorescence in *M. acetivorans* cells was examined during different growth phases. Levels of tdFAST2:HBR-3,5DOM fluorescence intensity differed significantly (Student's *t* test, *P* < 0.05) between cells harvested during exponential, late exponential, and stationary growth phase (Fig. 3). Here, fluorescence was most pronounced during late exponential growth phase, while it was at the lowest intensity during the stationary phase (Fig. 3). The *mcrB* promoter that controls tdFAST2 expression is considered a relatively strong constitutive promoter (18). Observed differences in tdFAST2:HBR-3,5DOM fluorescence might be due to general, growth phase-dependent fluctuations in protein accumulation or due to promoter-specific differences. In addition, autofluorescence was more intense (Student's *t* test, *P* < 0.05) during the late exponential growth phase than during the exponential and stationary growth phases (Fig. 3), which supports the hypothesis of growth phase-dependent differences in *M. acetivorans* cultures.

### Influence of tdFAST2 expression on cell growth.

**FIG 4:**
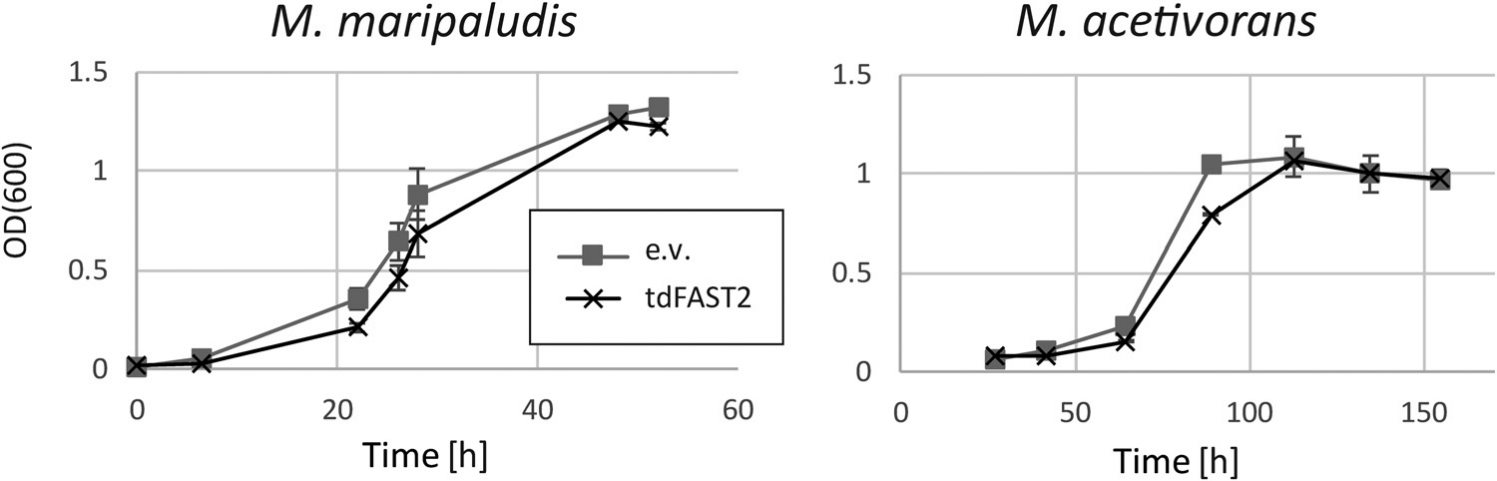
tdFAST2 is not toxic for methanogens. Growth of *M. acetivorans* and *M. maripaludis* expressing eighter tdFAST2 or harboring an empty vector construct (e.v.) was analyzed. Mean values and standard deviation of triplicates are shown.

An important point to consider when utilizing a reporter protein is whether there is any potential harmful or toxic effect on the cell. Therefore, we examined the general impact of tdFAST2 in methanogens. As shown in Fig. 4, tdFAST2 expression only led to a minor delay in cell growth, and thus, tdFAST2 can be considered as nontoxic for the methanogenic cells.

To study whether the HBR-3,5DOM fluorogen is cytotoxic, 5 μM fluorogen was added to growing *M. acetivorans* and *M. maripaludis* cultures. After 2 days, these cultures have produced a similar amount of methane (90% to 115%) compared to control cultures where no HBR-3,5DOM was added. Furthermore, turbidity of HBR-3,5DOM-treated cultures increased over time and were used successfully to inoculate new cultures after 2 days. We conclude that the 5 μM HBR-3,5DOM is not toxic for growing *M. acetivorans* and *M. maripaludis* cultures.

### Detection of tdFAST2:HBR-3,5DOM fluorescence via flow cytometry.

**FIG 5:**
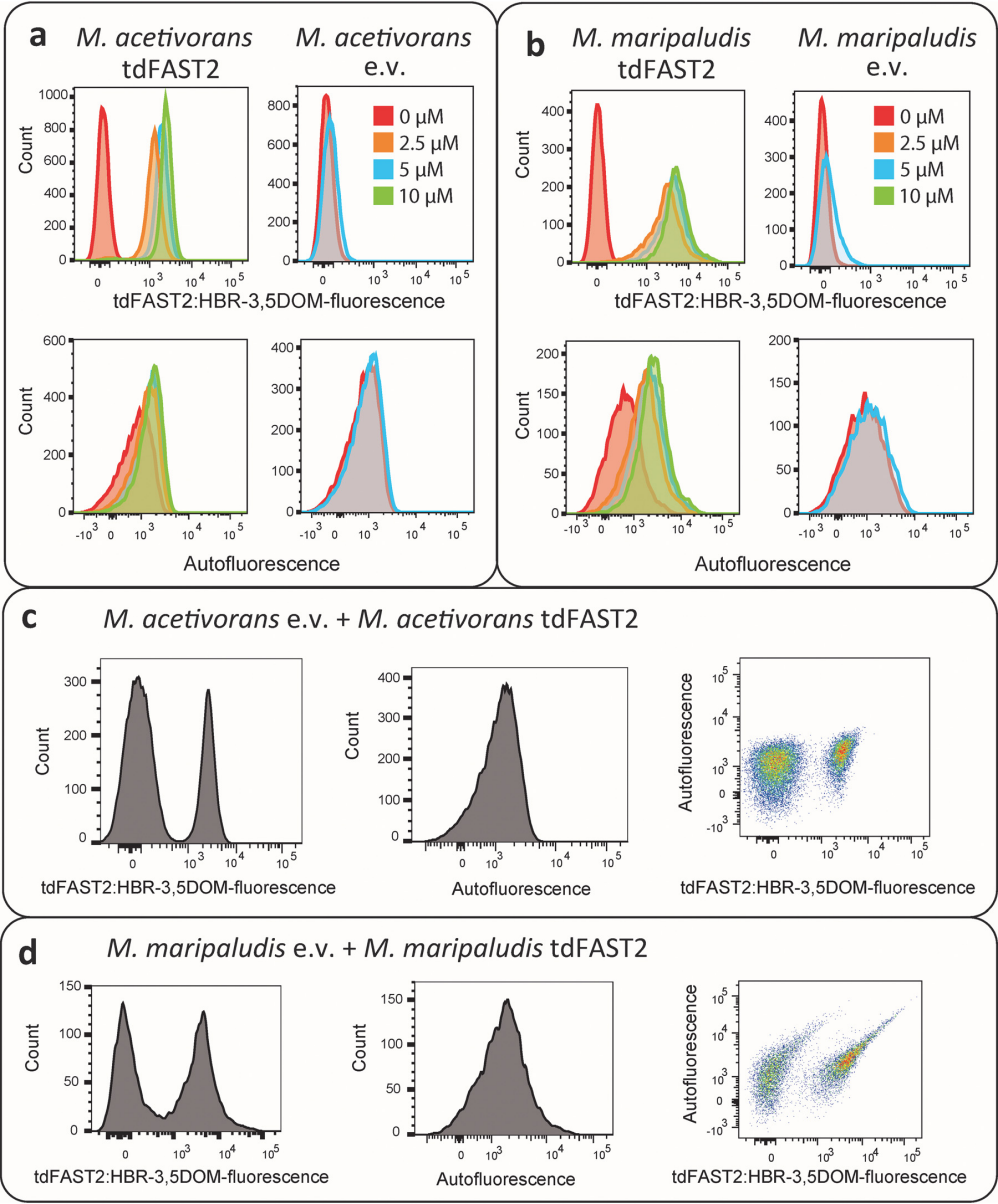
Flow cytometry allows visualization of tdFAST2 expression and separation of populations. Histograms of (a) *M. acetivorans* and (b) *M. maripaludis* cells expressing tdFAST2 (tdFAST2) or control cells carrying an empty vector construct (e.v.) in the presence of increasing fluorogen (HBR-3,5DOM) concentrations. (c and d) Mixtures of tdFAST2-expressing cells and non tdFAST2-expressing cells were analyzed in the presence of 5 mM HBR-3,5DOM. *M. acetivorans* cultures were mixed in a 2 to 1 ratio (excess of e.v.), while *M. maripaludis* cultures were mixed in a 1 to 1 ratio.

Measuring cellular fluorescence via flow cytometry is considered the gold standard for the detection of a fluorescent reporter. Therefore, we tested the feasibility of using flow cytometry to detect tdFAST2:HBR-3,5DOM fluorescence in methanogens. As shown in Fig. 5, tdFAST2:HBR-3,5DOM fluorescence was clearly detectable in tdFAST2-expressing *M. acetivorans* (Fig. 5a) and *M. maripaludis* (Fig. 5b) when the HBR-3,5DOM fluorogen was added. Fluorogen concentrations varied from 2.5 to 10 μM were used, which had a minor effect on the observed tdFAST2:HBR-3,5DOM fluorescence. This finding is consistent with measuring the tdFAST2:HBR-3,5DOM fluorescence using a microplate reader (Fig. 2b). F420-dependent autofluorescence was also measured. Here, a slight increase in autofluorescence was observed when cells exhibited tdFAST2:HBR-3,5DOM fluorescence signal. It is likely that autofluorescence detection also detects a certain amount of tdFAST2:HBR-3,5DOM fluorescence (Fig. 5a and b). When no tdFAST2 was expressed, fluorogen addition had no influence on the autofluorescence detected.

An interesting application for flow cytometry is to identify specific cell types within mixed populations. Thus, we used flow cytometry to measure fluorescence in *M. acetivorans* and *M. maripaludis* cultures in which tdFAST2-expressing and tdFAST2-nonexpressing cells were mixed together. Here, flow cytometry with mixed cultures clearly identified two tdFAST2:HBR-3,5DOM fluorescence peaks (Fig. 5c and d). For this, *M. acetivorans* cultures (Fig. 5c) were mixed in a 2 to 1 ratio with an excess of the empty vector-harboring culture, while *M. maripaludis* culture mixes (Fig. 5d) were used at a 1 to 1 ratio. As expected, a single peak was detected for autofluorescence. Similar results were obtained when the experiment was repeated with new culture mixes on different days (Fig. S3). Consequently, flow cytometry using tdFAST2:HBR-3,5DOM fluorescence-based detection is clearly able to distinguish and separate tdFAST2-expressing cells from tdFAST2-nonexpressing cells, although about 2% of the mixed culture population was not clearly identified as one or another of the different cell types.

### Construction of Golden Gate cloning vectors for tdFAST2-tagged proteins.

**FIG 6:**
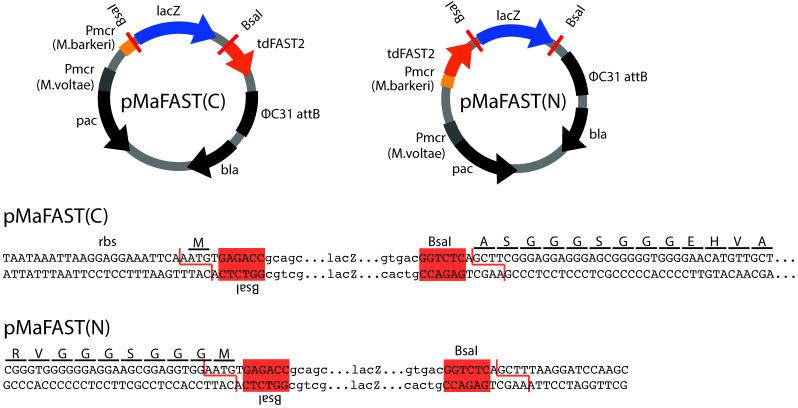
Golden Gate cloning vectors to construct tdFAST2-tagged proteins. The plasmids pMaFAST(C) and pMaFAST(N) encode the tdFAST2 gene which is codon-optimized for expression in *Methanosarcina*. Golden Gate cloning (*Bsa*I) can be used to replace the *lacZ* cassette with a protein-encoding sequence of interest leading to a C- or N-terminal fusion to the tdFAST2. Nucleotide sequence of the cloning sites are shown below. The *Bsa*I sites, ribosome-binding site (rbs) and the tdFAST2 open-reading frame is indicated.

To simplify the use of tdFAST2 as a fluorescence reporter in *M. acetivorans*, expression vectors that allow N- or C-terminal tdFAST2 fusions in a one-step Golden Gate cloning reaction (19, 20) were constructed. Here, the pNB730 plasmid was domesticated by removing *Bsa*I restriction sites in the vector backbone and the tdFAST2-encoding sequence was inserted. Additionally, a *lacZ* cassette was inserted to allow blue-white selection of positive Escherichia coli clones. The *lacZ* cassette is flanked by *Bsa*I sites and can be replaced by the protein sequence of interest. For cloning, the sequence of interest can be amplified with primers that add flanking *Bsa*I sites and the corresponding overhangs (AATG and AAGC) for insertion (forward primer overhang: 5′-TTTGGTCTCTAATG and reverse primer overhang: 5′-TTTGGTCTCTAAGC). If the inserted sequence is in frame with the tdFAST2 sequence, a AGGGSGGG (C-terminal tdFAST2) or GGGSGGGM (N-terminal tdFAST2) linker peptide is placed between tdFAST2 and the protein of interest (Fig. 6). The generated pMaFAST(C) and pMaFAST(N) plasmids contain a ΦC31 attB site, which allow for the integration into the *M. acetivorans* genome at attP sites, as well as the *pac* and *bla* genes needed for marker selection in *M. acetivorans* and E. coli, respectively.

## DISCUSSION

Here, we characterize tdFAST2:HBR-3,5DOM fluorescence as a reliable fluorescent reporter in two methanogenic model organisms, *M. acetivorans* and *M. maripaludis*. tdFAST2:HBR-3,5DOM fluorescence can be measure using a microplate reader or flow cytometer and can be distinguished from cellular autofluorescence, which can be measured in parallel. Importantly, tdFAST2 expression is nontoxic to the methanogens used. Further, the inclusion of a washing step was key to removing any background fluorescence due to the culture medium. This is in line with recent findings reporting strong HBR-3,5DOM-dependent background fluorescence occurring with *M. maripaludis* cultures (14). Likewise, a washing step also reduced background fluorescence from culture medium when FAST:fluorogen fluorescence was studied in anaerobic *Clostridium* (21).

### A versatile reporter system for methanogens.

To date, the β-glucuronidase gene (*uidA*) is the most frequently used reporter in methanogenic archaea. Here, β-glucuronidase activity is quantified by *in vitro* assays using cell extracts of *Methanosarcina* spp. (22, 23) and *Methanococcus* spp. (24). Additional but less commonly used enzymatic reporters in methanogens include acetohydroxyacid synthase, β-galactosidase, and β-lactamase (25, 26). However, enzymatic reporters have certain disadvantages: (i) the need for multiple handling steps that hinder high-throughput screenings, (ii) the inability to study single cells, and (iii) a relatively high cell culture volume requirement. These disadvantages were partly overcome studying *M. maripaludis* by using the mCherry fluorescent reporter protein (27, 28). While mCherry fluorescence can be easily monitored using a microplate reader and shows no overlap with cellular autofluorescence, its use is time-consuming due to cell lysis by freeze-thawing and overnight exposure to oxygen to allow for chromatophore maturation. The latest development of a new reporter system for methanogens is the quantification of protein accumulation in *M. maripaludis* cells using FAST1:HMBR fluorescence-based detection (14). Here, FAST1 was N-terminally fused to FruA, a hydrogenase subunit that is known to be more abundant during growth in a formate-containing medium than in a hydrogen-containing one. FAST1-FruA abundance in formate-grown and hydrogen-grown cells was quantified by anoxic microscopy, such that the FAST1:HMBR fluorescence of hundreds of single cells was monitored. Quantification of fluorescence intensities of several hundred single cells via microscopy can be time-consuming and impractical. FAST1:HMBR fluorescence in FAST1-FruA-expressing cultures could not be quantified using a microplate reader, likely due to the high level of cellular autofluorescence. By employing tdFAST2:HBR-3,5DOM fluorescence to study promoter activity or protein accumulation in *M. acetivorans* and *M. maripaludis* cells, results can be obtained without excessive handling and/or incubation time of other reporter systems (see above). Further, the use of flow cytometry can efficiently achieve an accurate quantification of single-cell fluorescence levels.

### Aerobic versus anaerobic detection of FAST:fluorogen fluorescence.

For technical reasons, we were only able to perform fluorescence measurements under aerobic conditions. Similarly, measurements for most enzymatic reporter systems are also performed aerobically. Moreover, the use of mCherry in *M. maripaludis* even required overnight exposure to oxygen prior to protein quantification (see above). To quantify tdFAST2:HBR-3,5DOM fluorescence signals, cells from anaerobic cultures were harvested, pelleted, washed, and subsequently analyzed using a plate reader or flow cytometer. The handling time before taking the fluorescence measurements was considered minimal, only 5 to 10 min. As prolonged exposure to oxygen (21 h) had only a minor effect on tdFAST2:HBR-3,5DOM fluorescence (Fig. S2), this indicates that tdFAST2 and HBR-3,5DOM fluorogen are highly stable. On the other hand, cellular autofluorescence showed a steep decrease after extensive exposure to oxygen (Fig. S2), which might possibly due to a low F420 stability. Despite the stability of tdFAST2 and HBR-3,5DOM, oxygen still has determinantal effects on methanogen physiology that likely results in rapid cell death. Nonetheless, we recognize that it is often unnecessary to use anoxic conditions when studying tdFAST2:fluorogen fluorescence in anaerobic methanogens. In fact, there is precedence in the published literature for analyzing FAST:fluorogen fluorescence in anaerobic organisms under aerobic conditions. For instance, the FAST:HMBR fluorescence of anaerobic *Clostridium* was determined aerobically using confocal microscopy as well as by taking microplate reader and flow cytometry measurements (21). Another recent study used flow cytometry aerobically to detect fluorescence of anaerobic acetogenic bacteria (29). Yet, in other studies oxygen is avoided when detecting the FAST:fluorogen fluorescence of anaerobic organisms, e.g., by placing the fluorescence microscope or microplate reader in an anaerobic chamber (14, 30). To the best of our knowledge, there are no reports in the literature of any major differences existing between the anoxic and oxic detection of FAST:fluorogen fluorescence in anaerobic organisms. We hypothesize that, although oxygen has a severe negative impact on the physiology of strictly anaerobic organisms, its short-term effect on protein abundance, protein localization, and protein-protein interaction is negligible. This assumption awaits further experimental confirmation in future studies.

### Applications of tdFAST2:HBR-3,5DOM fluorescence in methanogens.

There are many applications for the use of tdFAST2:HBR-3,5DOM fluorescence, including *in vivo* protein localization as well as the analysis of *in vivo* protein-protein interactions, in which the FAST2 protein is split and fused to different proteins of interest. Recently, both of these applications were successfully performed in *M. maripaludis* cells using FAST-fluorogen fluorescence-based detection (14). Flow cytometry facilitates the high-throughput measurement of various physical and biochemical characteristics of cells (31). The use of flow cytometry to detect and sort large quantities of individual cells based on tdFAST2:HBR-3,5DOM fluorescence will open new avenues for studying and engineering methanogens. For example, differential fluorescence induction (DFI) strategies (32) can be used to identify inducible promoters in *Methanosarcina* and *Methanococcus* species. By screening of a complex promoter library could be performed by cloning the different promoters in front of the tdFAST2 gene and then transforming into methanogens. The transformed culture could subsequently be treated with a particular inducer of gene expression, and thereafter individual cells would be screened and sorted for high tdFAST2:HBR-3,5DOM fluorescence via fluorescence-activated cell sorting (FACS). To eliminate constitutive promoters, cells with a high tdFAST2:HBR-3,5DOM fluorescence could be re-grown and sorted without the inducer. This approach requires to perform cell sorting under anaerobic conditions so that methanogen viability is maintained, a particular challenge that was already solved (33–36).The DFI strategy was successfully used with various bacteria, including Salmonella, Streptococcus, Pseudomonas, and *Bacillus* species (32).

Another application of flow cytometry that uses tdFAST2:HBR-3,5DOM fluorescence is to study population dynamics and responses in mixed methanogen cultures. Currently, the typical molecular characterization of *M. acetivorans* and *M. maripaludis* involves genetic modification (deletion or insertion) and then making comparisons between the modified cells to the wild type. For this, modified and wild-type cells are usually separated and characterized individually. Expression of tdFAST2 in either the wild-type or modified cells would allow the study of mixed cultures where tdFAST2:HBR-3,5DOM fluorescence is used to identify both populations. This real-time investigation of dynamics within mixed cultures would allow conclusions about the modification’s relevance for the organism. Measuring population dynamics in mixed culture also gains more relevance in biotechnological production where mixed cultures are increasingly used (37). A variety of methanogens are known to be involved in direct interspecies electron transfer (DIET) in coculture with electron-donating bacteria (38, 39). Here, the ability to distinguish and separate two type of methanogens by using tdFAST2:HBR-3,5DOM fluorescence might become an applicable tool to study DIET cocultures.

## MATERIALS AND METHODS

### Strains, cultivation, and transformation.

The strains *M. acetivorans* WWM73 (18) and *M. maripaludis* S0001 (40) were used throughout this study. Both methanogens were cultivated under strictly anaerobic conditions. For *M. acetivorans* growth, high salt (HS) medium (41) was used containing either 125 mM methanol (MeOH) or 50 mM trimethylamine (TMA) as the growth substrate. *M. maripaludis* was cultivated using McFC medium (42). PEG-mediated transformation (43, 44) was used for plasmid integration into methanogenic cells and 2 μg/mL puromycin was used as the selection marker when required. Plasmids pNB730::tdFAST2 and pNB730 were transformed in *M. acetivorans* and plasmids pMEV4::tdFAST2 and pMEV4 were transformed in *M. maripaludis*.

For plasmid construction, TOP10 E. coli strain (Thermo Fisher Scientific) was used with standard methods and enzymes were purchased from Thermo Fisher Scientific or New England BioLabs (NEB).

### Cloning of tdFAST2.

DNA sequences for the tdFAST2 (also known as td-iFAST [15]) gene were codon optimized using the Eurofins GENEius codon-optimization tool (www.geneius.de) and the *M. maripaludis* S2 and *M. acetivorans* C2A codon usage tables (http://www.kazusa.or.jp/codon/). A GGGSGGG linker peptide connecting the two FAST2 domains was used and internal restriction sites (*Bsa*I, *Bbs*I, and *Msm*BI) were removed. For expression in *M. maripaludis*, a ribosome-binding site (AGTGGGAGGTGCGC) and the transcriptional terminator from MMP1100 (AAATTCTTCTTCTTTTAAACGTTCTCCAGT [45]) was attached to the tdFAST2-coding sequence. For cloning, above-mentioned sequences were flanked by NdeI and BamHI sites or SpeI and PstI sites, respectively. DNA sequences were synthesized by Eurofins Scientific (Supplementary file 1). Classical cloning was used to clone the synthesized DNA fragments into pNB730 (46) for expression in *M. acetivorans* and pMEV4 (47) for expression in *M. maripaludis*. The resulting plasmids were named pNB730::tdFAST2 and pMEV4::tdFAST2, respectively.

### Fluorescence measurements.

For microplate reader measurements, a BioTek Cytation 3 Microplate Reader was used. Cells were harvested from anaerobic cultures and the optical density at 600 nm (OD600) was determined using a spectrophotometer. Cell pellets were obtained by centrifugation (11,000 g, 2 min) and resuspended in 1 mL of a salt solution (400 mM NaCl, 13 mM KCl, 54 mM MgCl_2_, 2 mM CaCl_2_) mimicking the HS medium. The high salt content serves to prevent cell lysis due to osmotic changes. Resuspended cells were split into two equal aliquots (500 μL) and the cells were pelleted again by centrifugation (11,000 g for 2 min). One cell pellet was resuspended in salt solution (see above) while the other cell pellet was resuspended in a salt solution containing the HBR-3,5DOM fluorogen. Volumes used to resuspend the cell pellets were adjusted to obtain a cell concentration of 1 OD(600) unit/mL. A fluorogen concentration of 5 μM was used, unless stated otherwise. A total of 100 μL aliquots were analyzed in Nunc 96-well flat-bottom microplates. Salt solution (see above) was used to determine background fluorescence values that were then subtracted from the fluorescence intensity emitted by the methanogenic cells. Background fluorescence typically ranged from 500 to 800 a.u. for autofluorescence settings (λ_Ex_ = 420 nm/λ_Em_ = 480 nm) and about 1 order of magnitude lower for tdFAST2:HBR-3,5DOM fluorescence settings (λ_Ex_ = 515 nm/λ_Em_ = 590 nm).

Flow cytometry measurements were performed with a LSRFortessa Cell Analyzer (BD Biosciences) at the HiLife Flow Cytometry Unit, University of Helsinki. Results were analyzed using the FlowJo^T^ v10.8 Software (BD Biosciences). Cells were harvested from anaerobic cultures, pelleted by centrifugation (11,000 g, 2 min), and resuspended in a salt solution (see above). The HBR-3,5DOM fluorogen (15) was added just prior to the flow cytometry analysis. For tdFAST2:HBR-3,5DOM fluorescence, a blue laser (488 nm excitation) and 610/20 nm filter were used, whereas for autofluorescence, a violet laser (405 nm excitation) and 510/50 nm filter were used. A total of 20,000 events were recorded.

HBR-3, (5DOM) and HMBR are derivatives of HBR (16). Both fluorogens were purchased from Twinkle Bioscience (www.the-twinkle-factory.com) and stored as a 5 mM stock solution in DMSO at –20°C.

### Construction of golden gate cloning vectors.

The pNB730 plasmid was used to construct the Golden Gate cloning destination vectors pMaFAST(C) and pMaFAST(N) as outlined in the protocol “Accommodating a Vector to Golden Gate Cloning” (19). For this, the *lacZ* cassette was amplified from pUC19 (New England Biolabs, Cat. no. N3041S) using the primers oNA311 (5′-ttgaagacaaAATGtgagaccgcagctggcacgacaggtttc) and oNA312 (5′-ttgaagacaaAAGCtgagaccgtcacagcttgtctgtaagcg). Primer overhangs include *Bpi*I restriction sites (5′-gaagac), 4-nt fusion sites, and *Bsa*I sites (5′-ggtctc) in reverse complementary orientation to the *lacZ* fragment. For the construction of pMaFAST(C), the vector backbone was amplified in two parts from pNB730::tdFAST2 to remove an internal *Bsa*I site. For this, two flanking primer pairs were used: oNA323 (5′-ttgaagacaaGCTTTAAgGATCCAAGCTTGGGCCCTCG)/oNA304 (5′-ttgaagacaaACGCTCACCGGCTCCAGATTTATC) and oNA305 (5′-ttgaagacaaGCGTGGATCTCGCGGTATCATTG)/oNA328 (5′-ttgaagacaaCATTCCACCtCCGCTtCCTCCcCCCACCCGTTTTACAAACACCCAGTAAC). For generation of pMaFAST(N), the vector backbone was also amplified in two parts from pNB730::tdFAST2 using the primer pairs oNA343 (5′-ttgaagacaaGCTTCGGGAGGGAGCGGGGGTGGGGAACACGTCGCGTTTGGCTC)/oNA304 and oNA305/oNA318 (5′-ttgaagacaaCATTTGAATTTCCTCCTTAATTTATTAAAATCATTTTGGGAC). Primers contain *Bpi*I restriction sites, along with specific 4-nt fusion sites to be compatible to each other upon ligation. Two backbone fragments and the *lacZ* fragment were fused together in a Golden Gate cloning reaction using *Bpi*I. The cloning product was transformed into E. coli and plated on ampicillin- and X-gal-containing LB plates. Blue colonies were selected and verified by DNA sequencing of the regions near the *Bsa*I cloning sites.

## References

[1] Conrad R. 2009. The global methane cycle: recent advances in understanding the microbial processes involved. Environ Microbiol Rep 1:285–292. 10.1111/j.1758-2229.2009.00038.x. 23765881

[2] Sengupta K, Pal S. 2021. A review on microbial diversity and genetic markers involved in methanogenic degradation of hydrocarbons: futuristic prospects of biofuel recovery from contaminated regions. Environ Sci Pollut Res Int 28:40288–40307. 10.1007/s11356-021-13666-3. 33844144

[3] Enzmann F, Mayer F, Rother M, Holtmann D. 2018. Methanogens: biochemical background and biotechnological applications. AMB Expr 8:1–22. 10.1186/s13568-017-0531-x. PMC5754280 29302756

[4] Bhatia SK, Bhatia RK, Jeon JM, Kumar G, Yang YH. 2019. Carbon dioxide capture and bioenergy production using biological system – A review. Renew Sustain Energy Rev 110:143–158. 10.1016/j.rser.2019.04.070.

[5] Zabranska J, Pokorna D. 2018. Bioconversion of carbon dioxide to methane using hydrogen and hydrogenotrophic methanogens. Biotechnol Adv 36:707–720. 10.1016/j.biotechadv.2017.12.003. 29248685

[6] Zhang S, Jiang J, Wang H, Li F, Hua T, Wang W. 2021. A review of microbial electrosynthesis applied to carbon dioxide capture and conversion: the basic principles, electrode materials, and bioproducts. J CO2 Util 51:101640. 10.1016/j.jcou.2021.101640.

[7] Leigh JA, Albers SV, Atomi H, Allers T. 2011. Model organisms for genetics in the domain Archaea: methanogens, halophiles, thermococcales and sulfolobales. FEMS Microbiol Rev 35:577–608. 10.1111/j.1574-6976.2011.00265.x. 21265868

[8] Nayak DD, Metcalf WW. 2018. Genetic techniques for studies of methyl-coenzyme M reductase from Methanosarcina acetivorans C2A. Methods Enzymol, 1st Ed 613:325–347. 10.1016/bs.mie.2018.10.012. 30509472

[9] Mondorf S, Deppenmeier U, Welte C. 2012. A novel inducible protein production system and neomycin resistance as selection marker for Methanosarcina mazei. Archaea 2012:973743. 10.1155/2012/973743. 22851906 PMC3407599

[10] Remington SJ. 2006. Fluorescent proteins: maturation, photochemistry and photophysics. Curr Opin Struct Biol 16:714–721. 10.1016/j.sbi.2006.10.001. 17064887

[11] Eirich LD, Vogels GD, Wolfe RS. 1978. Proposed structure for coenzyme F420 from methanobacterium. Biochemistry 17:4583–4593. 10.1021/bi00615a002. 728375

[12] Streett H, Charubin K, Papoutsakis ET. 2021. Anaerobic fluorescent reporters for cell identification, microbial cell biology and high-throughput screening of microbiota and genomic libraries. Curr Opin Biotechnol 71:151–163. 10.1016/j.copbio.2021.07.005. 34375813

[13] Chia HE, Marsh ENG, Biteen JS. 2019. Extending fluorescence microscopy into anaerobic environments. Curr Opin Chem Biol 51:98–104. 10.1016/j.cbpa.2019.05.008. 31252372

[14] Hernandez E, Costa KC. 2022. The fluorescence-activating and absorption-shifting tag (FAST) enables live-cell fluorescence imaging of Methanococcus maripaludis. J Bacteriol 204:7. 10.1128/jb.00120-22. PMC9295556 35657707

[15] Tebo AG, Pimenta FM, Zhang Y, Gautier A. 2018. Improved chemical-genetic fluorescent markers for live cell microscopy. Biochemistry 57:5648–5653. 10.1021/acs.biochem.8b00649. 30204425

[16] Plamont M-A, Billon-Denis E, Maurin S, Gauron C, Pimenta FM, Specht CG, Shi J, Quérard J, Pan B, Rossignol J, Moncoq K, Morellet N, Volovitch M, Lescop E, Chen Y, Triller A, Vriz S, Le Saux T, Jullien L, Gautier A. 2016. Small fluorescence-activating and absorption-shifting tag for tunable protein imaging in vivo. Proc Natl Acad Sci USA 113:497–502. 10.1073/pnas.1513094113. 26711992 PMC4725535

[17] Tebo AG, Gautier A. 2019. A split fluorescent reporter with rapid and reversible complementation. Nat Commun 10:1–8. 31249300 10.1038/s41467-019-10855-0 PMC6597557

[18] Guss AM, Rother M, Zhang JK, Kulkkarni G, Metcalf WW. 2008. New methods for tightly regulated gene expression and highly efficient chromosomal integration of cloned genes for Methanosarcina species. Archaea 2:193–203. 10.1155/2008/534081. 19054746 PMC2685592

[19] Marillonnet S, Grützner R. 2020. Synthetic DNA assembly using Golden Gate cloning and the hierarchical modular cloning pipeline. Curr Protoc Mol Biol 130:1–33. 10.1002/cpmb.115 32159931

[20] Engler C, Kandzia R, Marillonnet S. 2008. A one pot, one step, precision cloning method with high throughput capability. PLoS One 3:E3647. 10.1371/journal.pone.0003647. 18985154 PMC2574415

[21] Streett HE, Kalis KM, Papoutsakis ET. 2019. A strongly fluorescing anaerobic reporter and protein-tagging system for clostridium organisms based on the fluorescence-activating and absorption-shifting tag protein (FAST). Appl Environ Microbiol 85:1–15. 10.1128/AEM.00622-19. PMC6606882 31076434

[22] Pritchett MA, Zhang JK, Metcalf WW. 2004. Development of a markerless genetic exchange method for Methanosarcina acetivorans C2A and its use in construction of new genetic tools for methanogenic Archaea. Appl Environ Microbiol 70:1425–1433. 10.1128/AEM.70.3.1425-1433.2004. 15006762 PMC368415

[23] Guss AM, Kulkarni G, Metcalf WW. 2009. Differences in hydrogenase gene expression between Methanosarcina acetivorans and Methanosarcina barkeri. J Bacteriol 191:2826–2833. 10.1128/JB.00563-08. 19201801 PMC2668380

[24] Beneke S, Bestgen H, Klein A. 1995. Use of the Escherichia coli uidA gene as a reporter in Methanococcus voltae for the analysis of the regulatory function of the intergenic region between the operons encoding selenium-free hydrogenases. Mol Gen Genet 248:225–228. 10.1007/BF02190804. 7651345

[25] Gardner WL, Whitman WB. 1999. Expression vectors for Methanococcus maripaludis: overexpression of acetohydroxyacid synthase and β-galactosidase. Genetics 152:1439–1447. 10.1093/genetics/152.4.1439. 10430574 PMC1460687

[26] Demolli S, Geist MM, Weigand JE, Matschiavelli N, Suess B, Rother M. 2014. Development of β-lactamase as a tool for monitoring conditional gene expression by a tetracycline-riboswitch in Methanosarcina acetivorans. Archaea 2014:725610–725610. 24678266 10.1155/2014/725610 PMC3942078

[27] Akinyemi TS, Shao N, Lyu Z, Drake IJ, Liu Y, Whitman WB. 2021. Tuning gene expression by phosphate in the methanogenic archaeon Methanococcus maripaludis. ACS Synth Biol 10:3028–3039. 10.1021/acssynbio.1c00322. 34665610

[28] Lyu Z, Shao N, Chou CW, Shi H, Patel R, Duin EC, Whitman WB. 2020. Posttranslational methylation of arginine in methyl coenzyme M reductase has a profound impact on both methanogenesis and growth of Methanococcus maripaludis. J Bacteriol 202:1–18. 10.1128/JB.00654-19. PMC6964740 31740491

[29] Flaiz M, Baur T, Gaibler J, Kröly C, Dürre P. 2022. Establishment of green- and red- fluorescent reporter proteins based on the fluorescence-activating and absorption- shifting tag for use in acetogenic and solventogenic anaerobes. ACS Synth Biol 11:953–967. 10.1021/acssynbio.1c00554. 35081709

[30] Flaiz M, Ludwig G, Bengelsdorf FR, Dürre P. 2021. Production of the biocommodities butanol and acetone from methanol with fluorescent FAST-tagged proteins using metabolically engineered strains of Eubacterium limosum. Biotechnol Biofuels 14:1–20. 10.1186/s13068-021-01966-2. 33971948 PMC8111989

[31] Tracy BP, Gaida SM, Papoutsakis ET. 2010. Flow cytometry for bacteria: enabling metabolic engineering, synthetic biology and the elucidation of complex phenotypes. Curr Opin Biotechnol 21:85–99. 10.1016/j.copbio.2010.02.006. 20206495

[32] Rediers H, Rainey PB, Vanderleyden J, De Mot R. 2005. Unraveling the secret lives of bacteria: use of in vivo expression technology and differential fluorescence induction promoter traps as tools for exploring niche-specific gene expression. Microbiol Mol Biol Rev 69:217–261. 10.1128/MMBR.69.2.217-261.2005. 15944455 PMC1197422

[33] Thompson AW, Crow MJ, Wadey B, Arens C, Turkarslan S, Stolyar S, Elliott N, Petersen TW, van den Engh G, Stahl DA, Baliga NS. 2015. A method to analyze, sort, and retain viability of obligate anaerobic microorganisms from complex microbial communities. J Microbiol Methods 117:74–77. 10.1016/j.mimet.2015.07.009. 26187776

[34] Qi X, Carberry DM, Cai C, Hu S, Yuan Z, Rubinsztein-Dunlop H, Guo J. 2017. Optical sorting and cultivation of denitrifying anaerobic methane oxidation archaea. Biomed Opt Express 8:934. 10.1364/BOE.8.000934. 28270994 PMC5330591

[35] Bellais S, Nehlich M, Ania M, Duquenoy A, Mazier W, van den Engh G, Baijer J, Treichel NS, Clavel T, Belotserkovsky I, Thomas V. 2022. Species-targeted sorting and cultivation of commensal bacteria from the gut microbiome using flow cytometry under anaerobic conditions. Microbiome 10:1–17. 10.1186/s40168-021-01206-7. 35115054 PMC8812257

[36] Hamilton-Brehm SD, Vishnivetskaya TA, Allman SL, Mielenz JR, Elkins JG. 2012. Anaerobic high-throughput cultivation method for isolation of thermophiles using biomass-derived substrates. Methods Mol Biol 908:153–168. 10.1007/978-1-61779-956-3_15. 22843398

[37] Schlembach I, Grünberger A, Rosenbaum MA, Regestein L. 2021. Measurement techniques to resolve and control population dynamics of mixed-culture processes. Trends Biotechnol 39:1093–1109. 10.1016/j.tibtech.2021.01.006. 33573846 PMC7612867

[38] Wang W, Lee DJ. 2021. Direct interspecies electron transfer mechanism in enhanced methanogenesis: a mini-review. Bioresour Technol 330:124980. 10.1016/j.biortech.2021.124980. 33743275

[39] Barua S, Dhar BR. 2017. Advances towards understanding and engineering direct interspecies electron transfer in anaerobic digestion. Bioresour Technol 244:698–707. 10.1016/j.biortech.2017.08.023. 28818798

[40] Walters AD, Smith SE, Chong JPJ. 2011. Shuttle vector system for Methanococcus maripaludis with improved transformation efficiency. Appl Environ Microbiol 77:2549–2551. 10.1128/AEM.02919-10. 21296937 PMC3067461

[41] Sowers KR, Boone JE, Gunsalus RP. 1993. Disaggregation of Methanosarcina spp. and growth as single cells at elevated osmolarity. Appl Environ Microbiol 59:3832–3839. 10.1128/aem.59.11.3832-3839.1993. 16349092 PMC182538

[42] Long F, Wang L, Lupa B, Whitman WB. 2017. a flexible system for cultivation of methanococcus and other formate-utilizing methanogens. Archaea 2017:7046026. 10.1155/2017/7046026. 29348732 PMC5733999

[43] Oelgeschläger E, Rother M. 2009. In vivo role of three fused corrinoid/methyl transfer proteins in Methanosarcina acetivorans. Mol Microbiol 72:1260–1272. 10.1111/j.1365-2958.2009.06723.x. 19432805

[44] Tumbula DL, Makula RA, Whitman WB. 1994. Transformation of Methanococcus maripaludis and identification of a Pst I-like restriction system. FEMS Microbiol Lett 121:309–314. 10.1111/j.1574-6968.1994.tb07118.x.

[45] Yue L, Li J, Zhang B, Qi L, Li Z, Zhao F, Li L, Zheng X, Dong X. 2020. The conserved ribonuclease aCPSF1 triggers genome-wide transcription termination of archaea via a 3’-end cleavage mode. Nucleic Acids Res 48:9589–9605. 10.1093/nar/gkaa702. 32857850 PMC7515710

[46] Shea MT, Walter ME, Duszenko N, Ducluzeau AL, Aldridge J, King SK, Buan NR. 2016. PNEB193-derived suicide plasmids for gene deletion and protein expression in the methane-producing archaeon, Methanosarcina acetivorans. Plasmid 84–85:27–35. 10.1016/j.plasmid.2016.02.003. PMC4875793 26876941

[47] Lyu Z, Jain R, Smith P, Fetchko T, Yan Y, Whitman WB. 2016. Engineering the autotroph Methanococcus maripaludis for geraniol production. ACS Synth Biol 5:577–581. 10.1021/acssynbio.5b00267. 26886063

