# Supplementary material for: Application of the Fluorescence-Activating and Absorption-Shifting Tag (FAST) for Flow Cytometry in Methanogenic Archaea
Source: Appl Environ Microbiol. 2023 Mar 15;89(4):e01786-22. doi: 10.1128/aem.01786-22 (PMC10132111; doi:10.1128/aem.01786-22)
Supplement: Supplemental file 1 — Supplemental material. Download aem.01786-22-s0001.pdf, PDF file, 0.8 MB [file aem.01786-22-s0001.pdf]

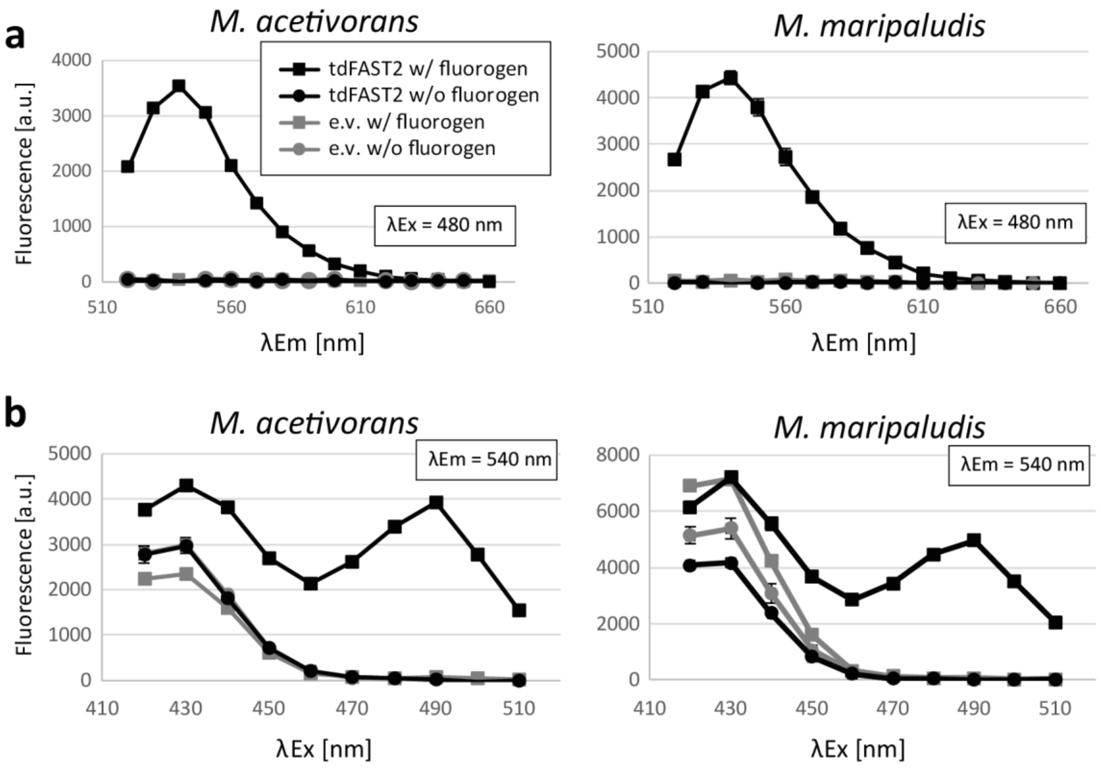

Supplementary Figure 1. tdFAST2-expressing methanogens show a specific fluorescent when the fluorogen HMBR is present.
Fluorescence of *M. acetivorans* and *M. maripaludis* cells is shown. **a**) Fluorescence spectrum upon excitation at 480 nm. **b**) Fluorescence at 540 nm when different excitation wavelengths are applied. Cells expressing tdFAST2 (tdFAST2) or control cells carrying an empty vector construct (e.v.) were analysed in presence (w/ fluorogen) or absence (w/o fluorogen) of HMBR. Used cells were in the stationary growth phase. Mean values and standard deviation of duplicates are shown.

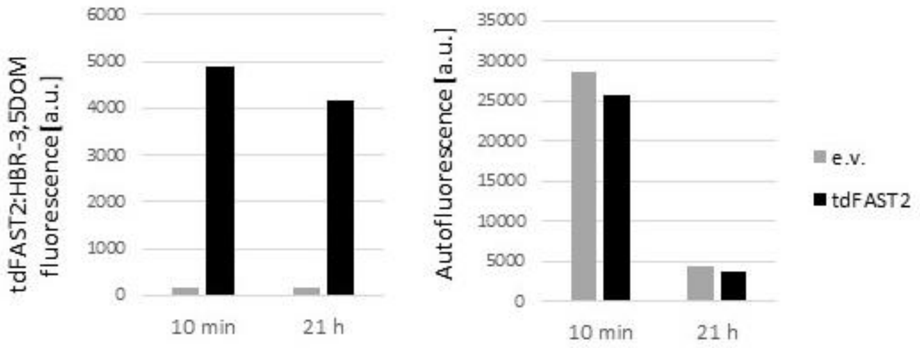

Supplementary Figure 2. tdFAST2:fluorogen fluorescence can be detected in methanogens after 21 h exposure to oxygen.

M. acetivorans cells expressing tdFAST2 or harbouring an empty vector construct (e.v.) were analyzed in the presence of 5 µM HBR-3,5DOM using a microplate reader. tdFAST2:HBR-3,5DOM fluorescence (λEx = 515 nm / λEm = 590 nm) and autofluorescence (λEx = 420 nm / λEm = 480 nm) was measured after storing the plate under aerobic conditions in the dark for 10 min and 21h.

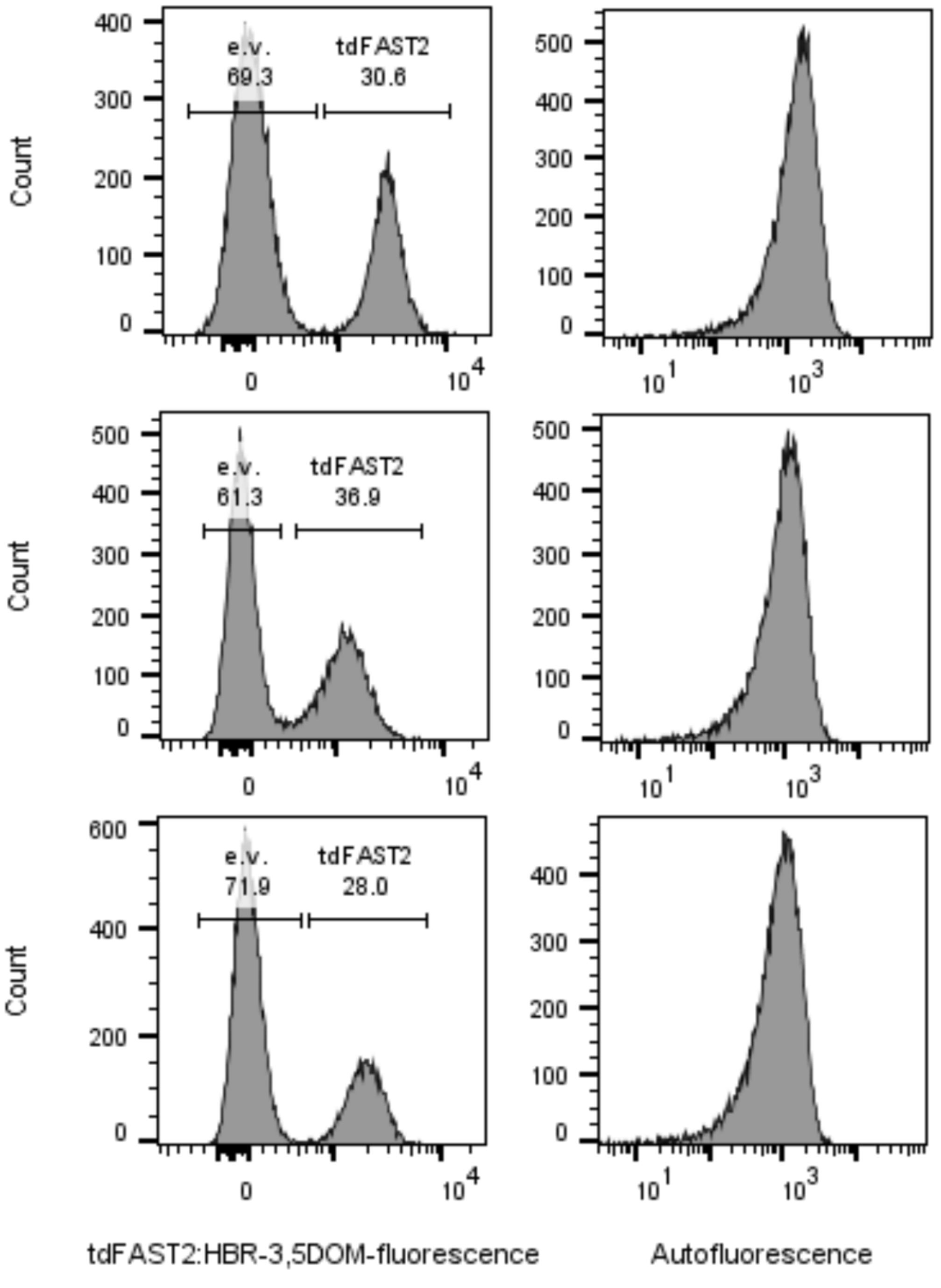

Supplementary Figure 3. Flow cytometry provides reproducible results. *M. acetivorans* cultures expressing tdFAST2 or harbouring an empty vector (e.v.) construct were mixed three times (top row, middle row, bottom row) from different cultures on different days in a ratio of 1:2 and analyzed with flow cytometry in the presence of 5 mM HBR-3,5DOM. Histograms show the HBR-3,5DOM-fluorescence and autofluorescence.

Supplementary File 1

Synthesized sequences

Synthesized fragment encoding codon-optimized tdFAST2 for expression in *M. acetivorans*

CATATGGAACACGTCGCGTTTGGCTCGGAAGATATTGAGAACACGTTAGCTAAAATGGAT
GATGGTCAACTAGACGGCTTAGCCTTCGGTGCAATACAGCTGGACGGAGATGGCAATATC
TTGCAGTATAATGCCGCGGAGGGCGACATAACCGGACGAGATCCTAAACAGGTCATTGGA
AAGAACTTCTTCAAGGATGTAGCCCCAGGCACCGATTCCCCTGAATTTTACGGAAAGTTC
AAAGAAGGAGTTGCAAGTGGGAACCTGAATACGATGTTTGAATGGATGATTCCGACCTCA
AGAGGTCCTACCAAAGTTAAGATACACATGAAAAAGGCCCTTTCCGGAGATTCTTATTGG
GTATTCGTTAAGAGGGTTGGAGGAGGGAGCGGGGGTGGGGAACATGTTGCTTTTGGAAGC
GAAGATATCGAAAACACCCTTGCAAAAATGGATGATGGACAACTCGATGGTCTGGCTTTT
GGCGCTATCCAGCTGGATGGGGACGGGAATATACTCCAGTACAATGCTGCAGAAGGAGAC
ATTACAGGAAGGGACCCAAAGCAGGTAATCGGCAAAAACTTCTTCAAAGACGTTGCACCA
GGAACGGACTCGCCAGAATTCTATGGGAAGTTTAAAGAAGGGGTCGCTTCCGGTAACCTT
AACACTATGTTTGAGTGGATGATTCCCACGTCTAGAGGTCCCACTAAAGTGAAAATACAC
ATGAAAAAAGCACTTAGTGGAGACAGTTACTGGGTGTTTGTAAAACGGGTGTAAGGATCC

underlined = tdFAST2 protein-encoding sequence

CATATG = *Nde*I restriction site

GGATCC =*Bam*HI *site*

Synthesized fragment encoding codon-optimized tdFAST2 for expression in *M. maripaludis*

ACTAGTAGTGGGAGGTGCGCTATGGAACATGTGGCCTTCGGATCAGAAGATATAGAGAAC
ACTTTAGCCAAGATGGATGATGGGCAATTAGACGGATTAGCGTTTGGGGCAATTCAGCTA
GATGGGGATGGTAACATTCTTCAATACAATGCTGCTGAAGGGGATATAACCGGGCGAGAT
CCAAAACAGGTAATTGGAAAGAATTTTTTCAAAGACGTCGCACCGGGTACAGATTCACCT
GAATTTTATGGTAAATTTAAAGAAGGTGTTGCAAGTGGAAATCTCAATACGATGTTCGAA
TGGATGATACCTACATCTAGAGGACCTACGAAAGTAAAAATCCACATGAAAAAAGCTCTA
TCAGGTGACAGCTATTGGGTATTTGTTAAAAGAGTAGGAGGCGGGTCAGGAGGAGGAGAA
CACGTCGCTTTCGGTAGTGAAGATATAGAAAACACATTAGCAAAGATGGACGACGGACAG
CTTGATGGGCTTGCATTTGGAGCAATCCAATTAGATGGAGATGGTAATATACTTCAATAC
AATGCAGCAGAAGGTGACATCACTGGTAGAGATCCCAAACAGGTCATTGGAAAAAATTTT
TTTAAAGATGTTGCACCAGGAACAGATTCCCCAGAATTTTACGGAAAATTTAAAGAAGGA
GTAGCATCTGGAAATTTGAATACAATGTTTGAATGGATGATTCCAACTTCTAGAGGACCA
ACTAAAGTTAAGATTCACATGAAAAAAGCACTGTCGGGAGATTCATATTGGGTTTTTGTT
AAAAGGGTTTAA**AAATTCTTCTTCTTTTAAACGTTCTCCAGT**CTGCAG

underlined = tdFAST2 protein-encoding sequence

bold = terminator sequence

ACTAGT = *Spe*I restriction site

CTGCAG =*Pst*I site

Supplementary File 2
Plasmid sequences

pNB730::tdFAST2
TCTAGTTGTCTGCTCCCGGCATCCGCTTACAGACAAGCTGTGACCGTCTCCGGGAGCTGCATGTGTCAGAG
GTTTTCACCGTCATCACCGAAACGCGCGAGACGAAAGGGCCTCGTGATACGCCTATTTTTATAGGTTAATGT
CATGATAATAATGGTTTCTTAGACGTCAGGTGGCACTTTTCGGGGAAATGTGCGCGGAACCCCTATTTGTTT
ATTTTTCTAAATACATTCAAATATGTATCCGCTCATGAGACAATAACCCTGATAAATGCTTCAATAATATTGA
AAAAGGAAGAGTATGAGTATTCAACATTTCCGTGTCGCCCTTATTCCCTTTTTTGCGGCATTTTGCCTTCCTG
TTTTTGCTCACCCAGAAACGCTGGTGAAAGTAAAAGATGCTGAAGATCAGTTGGGTGCACGAGTGGGTTAC
ATCGAACTGGATCTCAACAGCGGTAAGATCCTTGAGAGTTTTCGCCCCGAAGAACGTTTTCCAATGATGAGC
ACTTTTAAAGTTCTGCTATGTGGCGCGGTATTATCCCGTATTGACGCCGGGCAAGAGCAACTCGGTCGCCGC
ATACACTATTCTCAGAATGACTTGGTTGAGTACTCACCAGTCACAGAAAAGCATCTTACGGATGGCATGACA
GTAAGAGAATTATGCAGTGCTGCCATAACCATGAGTGATAACACTGCGGCCAACTTACTTCTGACAACGATC
GGAGGACCGAAGGAGCTAACCGCTTTTTTGCACAACATGGGGGATCATGTAACTCGCCTTGATCGTTGGGA
ACCGGAGCTGAATGAAGCCATACCAAACGACGAGCGTGACACCACGATGCCTGTAGCAATGGCAACAACG
TTGCGCAAACTATTAACTGGCGAACTACTTACTCTAGCTTCCCGGCAACAATTAATAGACTGGATGGAGGCG
GATAAAGTTGCAGGACCACTTCTGCGCTCGGCCCTTCCGGCTGGCTGGTTTATTGCTGATAAATCTGGAGCC
GGTGAGCGTGGGTCTCGCGGTATCATTGCAGCACTGGGGCCAGATGGTAAGCCCTCCCGTATCGTAGTTAT
CTACACGACGGGGAGTCAGGCAACTATGGATGAACGAAATAGACAGATCGCTGAGATAGGTGCCTCACTG
ATTAAGCATTGGTAACTGTCAGACCAAGTTTACTCATATATACTTTAGATTGATTTAAAACTTCATTTTTAATT
TAAAAGGATCTAGGTGAAGATCCTTTTTGATAATCTCATGACCAAAATCCCTTAACGTGAGTTTTCGTTCCAC
TGAGCGTCAGACCCCGTAGAAAAGATCAAAGGATCTTCTTGAGATCCTTTTTTTCTGCGCGTAATCTGCTGC
TTGCAAACAAAAAAACCACCGCTACCAGCGGTGGTTTGTTTGCCGGATCAAGAGCTACCAACTCTTTTTCCG
AAGGTAACTGGCTTCAGCAGAGCGCAGATACCAAATACTGTTCTTCTAGTGTAGCCGTAGTTAGGCCACCA
CTTCAAGAACTCTGTAGCACCGCCTACATACCTCGCTCTGCTAATCCTGTTACCAGTGGCTGCTGCCAGTGGC
GATAAGTCGTGTCTTACCGGGTTGGACTCAAGACGATAGTTACCGGATAAGGCGCAGCGGTCGGGCTGAA
CGGGGGGTTCGTGCACACAGCCCAGCTTGGAGCGAACGACCTACACCGAACTGAGATACCTACAGCGTGA
GCTATGAGAAAGCGCCACGCTTCCCGAAGGGAGAAAGGCGGACAGGTATCCGGTAAGCGGCAGGGTCGG
AACAGGAGAGCGCACGAGGGAGCTTCCAGGGGGAAACGCCTGGTATCTTTATAGTCCTGTCGGGTTTCGC
CACCTCTGACTTGAGCGTCGATTTTTGTGATGCTCGTCAGGGGGGCGGAGCCTATGGAAAAACGCCAGCAA
CGCGGCCTTTTTACGGTTCCTGGCCTTTTGCTGGCCTTTTGCTCACATGTTCTTTCCTGCGTTATCCCCTGATT
CTGTGGATAACCGTATTACCGCCTTTGAGTGAGCTGATACCGCTCGCCGCAGCCGAACGACCGAGCGCAGC
GAGTCAGTGAGCGAGGAAGCGGAAGAGCGCCCAATACGCAAACCGCCTCTCCCCGCGCGTTGGCCGATTC
ATTAATGCAGCTGGCACGACAGGTTTCCCGACTGGAAAGCGGGCAGTGAGCGCAACGCAATTAATGTGAG
TTAGCTCACTCATTAGGCACCCCAGGCTTTACACTTTATGCTTCCGGCTCGTATGTTGTGTGGAATTGTGAGC
GGATAACAATTTCACACAGGAAACAGCTATGACCATGATTACGCCAAGCTTGCATGCTCATGCTCCAGGTTT
CCTGTCCTTCGGGCACCTCGACGTCGGCGGTGACGGTGAAGCCGAGCCGCTCGTAGAAGGGGAGGTTGCG
GGGCGCGGAGGTCTCCAGGAAGGCGGGCACCCCGGCGCGCTCGGCCGCCTCCACTCCGGGGAGCACGAC
GGCGCTGCCCAGACCCTTGCCCTGGTGGTCGGGCGAGACGCCGACGGTGGCCAGGAACCACGCGGGCTCC
TTGGGCCGGTGCGGCGCCAGGAGGCCTTCCATCTGTTGCTGCGCGGCCAGCCGGGAACCGCTCAACTCGG
CCATGCGCGGGCCGATCTCGGCGAACACCGCCCCCGCTTCGACGCTCTCCGGCGTGGTCCAGACCGCCACC
GCGGCGCCGTCGTCCGCGACCCACACCTTGCCGATGTCGAGCCCGACGCGCGTGAGGAAGAGTTCTTGCA
GCTCGGTGACCCGCTCGATGTGGCGGTCCGGGTCGACGGTGTGGCGCGTGGCGGGGTAGTCGGCGAACG
CGGCGGCGAGGGTGCGTACGGCCCGGGGGACGTCGTCGCGGGTGGCGAGGCGCACCGTGGGTTTATATT
CGGTCATGAGAATCACTCTAGTTCCTATTTTTTTGATATATACATCATAACATTACTCTATGTATATATATTCA
CTTTTTCATTAACATTAAATAGAAAAGTTTATATATAAGATGTTAATAACACAATAATTTGAATTTGAATACT

CAAAAAATGGGCTTTAATATATAAAATTAAGATGAAAATAGATGATTTTTTAAAAAAATGTTATTATTATATC
TCAATATCTAAATATTAGATTAATATTAATTATTACCCAAATATTTCAATGAATATTTAGTTTTGAATAGTATA
TTACGAATAGGGCGTTTTTTATTACCTACTACTATTTTCCGAAGATTTTTTAAGACTCTCTTAAAATTAATCAT
CCTCTAGAGGCGCGCCAATACGCAAACCGACTAGACTTAATTAAGATCCGGCGCGCCCCCGGGTACCGAGC
TCGAATTCACTGGCCGTCGTTTTACAACGTCGTGACTGGGAAAACCCTGGCGTTACCCAACTTAATCGCCTT
GCAGCACATCCCCCTTTCGCCAGCTGGCGTAATAGCGAAGAGGCCCGCACCGATCGCCCTTCCCAACAGTT
GCGCAGCCTGAATGGCGAATGGCGCCTGATGCGGTATTTTCTCCTTACGCATCTGTGCGGTATTTCACACCT
CATTTATCGGAGAACACAAAAGATTTAAGTACCTTCTAAACGAATGAGATTTCATTGGGAATAGTGGACACT
CGAGTAGGTGACCAGTCCCAAAATGATTTTAATAAATTAAGGAGGAAATTCATATGGAACACGTCGCGTTT
GGCTCGGAAGATATTGAGAACACGTTAGCTAAAATGGATGATGGTCAACTAGACGGCTTAGCCTTCGGTGC
AATACAGCTGGACGGAGATGGCAATATCTTGCAGTATAATGCCGCGGAGGGCGACATAACCGGACGAGAT
CCTAAACAGGTCATTGGAAAGAACTTCTTCAAGGATGTAGCCCCAGGCACCGATTCCCCTGAATTTTACGGA
AAGTTCAAAGAAGGAGTTGCAAGTGGGAACCTGAATACGATGTTTGAATGGATGATTCCGACCTCAAGAG
GTCCTACCAAAGTTAAGATACACATGAAAAAGGCCCTTTCCGGAGATTCTTATTGGGTATTCGTTAAGAGGG
TTGGAGGAGGGAGCGGGGGTGGGGAACATGTTGCTTTTGGAAGCGAAGATATCGAAAACACCCTTGCAAA
AATGGATGATGGACAACTCGATGGTCTGGCTTTTGGCGCTATCCAGCTGGATGGGGACGGGAATATACTCC
AGTACAATGCTGCAGAAGGAGACATTACAGGAAGGGACCCAAAGCAGGTAATCGGCAAAAACTTCTTCAA
AGACGTTGCACCAGGAACGGACTCGCCAGAATTCTATGGGAAGTTTAAAGAAGGGGTCGCTTCCGGTAAC
CTTAACACTATGTTTGAGTGGATGATTCCCACGTCTAGAGGTCCCACTAAAGTGAAAATACACATGAAAAAA
GCACTTAGTGGAGACAGTTACTGGGTGTTTGTAAAACGGGTGTAAgGATCCAAGCTTGGGCCCTCGCGACT
CGAGACACCACCATCATCATCACCACCATTGGTCCCATCCCCAATTTGAAAAGTAGTTAATGACGCGCCCTG
ACGGGACTAGAATGAATCAACAACTCTCCTGGCGCACCATCGTCGGCTACAGCCTCGGTGACGTCGCCAAT
AACTTCGCCTTCGCAATGGGGGCGCTCTTCCTGTTGAGTTACTACACCGACGTCGCTGGCGTCGGTGCCGCT
GCGGCGGGCACCATGCTGTTACTGGTGCGGGTATTCGATGCCTTCGCCGACGTCTTTGCCGGACGAGTGGT
GGACAGTGTGAATATCCGCTGGGGAAAATTCCGCCCGTTTTTACTCTTCGGTACTGCGCCGTTAATGATCAG
ATCCGAGCTCAAGCTTCTTGATAACTTCGTATAATGTATGCTATACGAAGTTATCCCTTTAGTGAGGGTTAAT
TAAGCGGCCGCCCGGGCCGGCCATTTAAATGCATGCGACTTCCGAAAAAACAGCAAAGAAAAGCCAGTAT
GGAAAAAATAGACAAAAAGTAGGCTAAAAGGCCTACTCTGTTTTAAACTGTTGAATTTATTGAGTTCGAGT
GAGGTGGAGTACGCGCCCGGGGAGCCCAAGGGCACGCCCTGGCACCCGCACCGCGGATCGATCGAATTCT
CGACCAATTCTCATGTTTGACAGCTTATCATCGAATTTCTGCCATTCATCCGCTTATTATCACTTATTCAGGCG
TAGCAACCAGGCGTTTAAGGGCACCAATAACTGCCTTAAAAAAATTACGCCCCGCCCTGCCACTCATCGCAG
TACTGTTGTAATTCATTAAGCATTCTGCCGACATGGAAGCCATCACAAACGGCATGATGAACCTGAATCGCC
AGCG

pMEV4::tdFAST2

cgatgcctgtagcaatggcaacaacgttgcgcaaactattaactggcgaactacttactctagcttcccggcaacaattaatagactggatgg
aggcggataaagttgcaggaccacttctgcgctcggcccttccggctggctggtttattgctgataaatctggagccggtgagcgtgggtctcg
cggtatcattgcagcactggggccagatggtaagccctcccgtatcgtagttatctacacgacggggagtcaggcaactatggatgaacgaa
atagacagatcgctgagataggtgcctcactgattaagcattggtaactgtcagaccaagtttactcatatatactttagattgatttaaaactt
catttttaatttaaaaggatctaggtgaagatcctttttgataatctcatgaccaaaatcccttaacgtgagttttcgttccactgagcgtcagac
cccgtagaaaagatcaaaggatcttcttgagatcctttttttctgcgcgtaatctgctgcttgcaaacaaaaaaaccaccgctaccagcggtgg
tttgtttgccggatcaagagctaccaactctttttccgaaggtaactggcttcagcagagcgcagataccaaatactgttcttctagtgtagccg
tagttaggccaccacttcaagaactctgtagcaccgcctacatacctcgctctgctaatcctgttaccagtggctgctgccagtggcgataagt
cgtgtcttaccgggttggactcaagacgatagttaccggataaggcgcagcggtcgggctgaacggggggttcgtgcacacagcccagcttg
gagcgaacgacctacaccgaactgagatacctacagcgtgagctatgagaaagcgccacgcttcccgaagggagaaaggcggacaggtat
ccggtaagcggcagggtcggaacaggagagcgcacgagggagcttccagggggaaacgcctggtatctttatagtcctgtcgggtttcgcca
cctctgacttgagcgtcgatttttgtgatgctcgtcaggggggcggagcctatggaaaaacgccagcaacgcggcctttttacggttcctggcc

ttttgctggccttttgctcacatggtacccattatttagtttttttttatcaccacaacgtataaaccccctttcagaagaaaacagagaatgaaa
acagattttaatttatatatcgtatctataatttagtttttttttattgcagttttagaagtgcgtttgtcctggagcgaaatacacgatatcgtgta
gtctccttcagatgaaaataatatttttaattttcagaaatattactggaaaaatacctgaaaaatatttgaaaaaaaaacatacacccagcg
gggaaaaatcgaacattttgaaaaagtctatttatagggtttaaacagggccaagacctgcgggatataccccaaaaatgttcgataaaaaa
atcgaacatttttcgaacatttttataatgttcgatgtgtgcgagtatgttcgataaaaaaatcgaacattttgaaagacaatttgaagagata
taaaaaaaataatctattataatatagatatataatatgttcgatatgttcgatattttttatatattatattatatttaaccttgtattgattatt
gaataatatttattattactttttttgatttcttgtaaaaaatcgaacatattttacatattgtaaaaaataaaaaagatttaaaaaaagtgtccg
aaaaagttaaacaaacattattattaaaatgttagtttaatatgtatcttgatatgctcttctttctttcatgcttaaacttgtataactgtatgctt
ttttcatttcctttattttgtcctttcctttctttgttatctggtaatatccttccttctttcctgcatacttcaaaaatccaaggtttttaagttatcga
gataagaattactagatgaattcgcgcccggttctagacgagataagaattactagataattcagctgatcgatcaaaatataacataaata
acataggtttaaataatttaaaggcatattttatataaacaattgtaaaatattggcttatgaaatttgttaaaatttagctaataagctattgat
attataattttatagataactaataCTAGTagtgggaggtgcgctATGGAACATGTGGCCTTCGGATCAGAAGATATAGAG
AACACTTTAGCCAAGATGGATGATGGGCAATTAGACGGATTAGCGTTTGGGGCAATTCAGCTAGATGGGG
ATGGTAACATTCTTCAATACAATGCTGCTGAAGGGGATATAACCGGGCGAGATCCAAAACAGGTAATTGGA
AAGAATTTTTTCAAAGACGTCGCACCGGGTACAGATTCACCTGAATTTTATGGTAAATTTAAAGAAGGTGTT
GCAAGTGGAAATCTCAATACGATGTTCGAATGGATGATACCTACATCTAGAGGACCTACGAAAGTAAAAAT
CCACATGAAAAAAGCTCTATCAGGTGACAGCTATTGGGTATTTGTTAAAAGAGTAGGAGGCGGGTCAGGA
GGAGGAGAACACGTCGCTTTCGGTAGTGAAGATATAGAAAACACaTTAGCAAAGATGGACGACGGACAGC
TTGATGGGCTTGCATTTGGAGCAATCCAATTAGATGGAGATGGTAATATACTTCAATACAATGCAGCAGAA
GGTGACATCACTGGTAGAGATCCCAAACAGGTCATTGGAAAAAATTTTTTTAAAGATGTTGCACCAGGAAC
AGATTCCCCAGAATTTTACGGAAAATTTAAAGAAGGAGTAGCATCTGGAAATTTGAATACAATGTTTGAAT
GGATGATTCCAACtTCTAGAGGACCAACTAAAGTTAAGATTCACATGAAAAAAGCACTGTCGGGAGATTCAT
ATTGGGTTTTTGTTAAAAGGGTTtaaaaattcttcttcttttaaacgttctccagtctgcagataaaaaacgccctattcgtaatatac
tattcaaaactaaatattcattgaaatatttgggtaataattaatattaatctaatatttagatattgagatataataataacatttttttaaaaa
atcatctattttcatcttaattttatatattaaagcccatttttgagtattcaaattcaaattattgtgttattaacatcttatatataaacttttcta
tttaatgttaatgaaaaagtgaatatatatacatagagtaatgttatgatgtatatatcaaaaaaaaactagaggagacgaccttccatgacag
aatataaaccaacagttagattagcaacaagagatgacgtacctagggcagtaagaactcttgcagcagcttttgcagattatccagcaaca
agacacacagttgatccagatagacacattgaaagagttacagaattacaagaattatttttaacaagagttggtcttgatataggtaaagta
tgggttgcagatgatggagcagcagttgcagtttggacaacaccagaatcagttgaagctggtgctgtttttgcagaaattggaccaagaatg
gctgaattatcaggatcaagattagcagctcagcaacagatggaaggattacttgcaccacacagaccaaaagaaccagcatggtttttagc
aacagttggagtttcaccagatcaccagggaaaaggtttaggttctgctgttgtacttcctggtgttgaagcagctgaaagagcaggagttcc
agcatttttagaaacatcagcaccaagaaatcttccattttatgaaagacttggatttacagttacagcagatgttgaagttccagaaggacc
aagaacatggtgtatgacaagaaaaccaggagcataaccatggctccgaccgaagccacccggggcggccccgccgaccccgcacccgcc
cccgaggcccaccgcgggggacacaccgaacacgccgaccctgctgaacacgcggcgcagttcggtgcccaggagcggatcgggaattaa
ttcgaagctgctggtgaaagagaccctatcttacctgctaaaatctaagttaattactaatttattattaatttattattagattgggtaaaatag
taaaagaaaactaaaggaaacctaatatggtttcctttttttatatatttttaattcactgggggggatttaaaacttcatttttaatttaaaagga
tctaggtgaagatccttggtgcactctcagtacaatctgctctgatgccgcatagttaagccagccccgacacccgccaacacccgctgacgc
gccctgacgggcttgtctgctcccggcatccgcttacagacaagctgtgaccgtctccgggagctgcatgtgtcagaggttttcaccgtcatca
ccgaaacgcgcgagacgaaagggcctcgtgatacgcctatttttataggttaatgtcatgataataatggtttcttagacgtcaggtggcactt
ttcggggaaatgtgcgcggaacccctatttgtttatttttctaaatacattcaaatatgtatccgctcatgagacaataaccctgataaatgcttc
aataatattgaaaaaggaagagtatgagtattcaacatttccgtgtcgcccttattcccttttttgcggcattttgccttcctgtttttgctcaccc
agaaacgctggtgaaagtaaaagatgctgaagatcagttgggtgcacgagtgggttacatcgaactggatctcaacagcggtaagatccttg
agagttttcgccccgaagaacgttttccaatgatgagcacttttaaagttctgctatgtggcgcggtattatcccgtattgacgccgggcaaga
gcaactcggtcgccgcatacactattctcagaatgacttggttgagtactcaccagtcacagaaaagcatcttacggatggcatgacagtaag
agaattatgcagtgctgccataaccatgagtgataacactgcggccaacttacttctgacaacgatcggaggaccgaaggagctaaccgctt
ttttgcacaacatgggggatcatgtaactcgccttgatcgttgggaaccggagctgaatgaagccataccaaacgacgagcgtgacacca
